# Epigenetic regulation of gene expression by Ikaros, HDAC1 and Casein Kinase II in leukemia

**DOI:** 10.1038/leu.2015.331

**Published:** 2016-01-22

**Authors:** C Song, X Pan, Z Ge, C Gowda, Y Ding, H Li, Z Li, G Yochum, M Muschen, Q Li, K J Payne, S Dovat

**Affiliations:** 1 Department of Pediatrics, Pennsylvania State University Medical College, Hershey, PA, USA; 2 Department of Hematology, The First Affiliated Hospital of Nanjing Medical University, Jiangsu Province Hospital, Nanjing, China; 3 Jilin Province Animal Embryo Engineering Key Laboratory, College of Animal Science and Veterinary Medicine, Jilin University, Changchun, China; 4 Department of Biochemistry and Molecular Biology, Pennsylvania State University Medical College, Hershey, PA, USA; 5 University of California San Francisco, San Francisco, CA, USA; 6 Department of Statistics, Pennsylvania State University, University Park, State College, PA, USA; 7 Department of Pathology and Human Anatomy and Center for Health Disparities and Molecular Medicine, Loma Linda University, Loma Linda, CA, USA

*IKZF1* (*Ikaros*) encodes a DNA-binding protein that acts as a master regulatory of hematopoiesis and a tumor suppressor in acute lymphoblastic leukemia (ALL).^1, 2, 3, 4^ The deletion and/or mutation of *Ikaros* is associated with the development of B-cell acute lymphoblastic leukemia (B-ALL) with poor outcome.^5, 6, 7, 8, 9, 10, 11^ Ikaros directly associates with components of the histone deacetylase complex (NuRD), HDAC1, HDAC2 and Mi-2.^12, 13, 14^ Although Ikaros is hypothesized to regulate the transcription of target genes by recruiting the NuRD complex, the mechanism of Ikaros-mediated transcriptional regulation in leukemia is still unknown. Here we use a systems biology approach to determine the mechanism through which Ikaros and HDAC1 regulate gene expression in human B-ALL.

To study the role of Ikaros and Ikaros–HDAC1 complexes in ALL, we determined the genome-wide occupancy of Ikaros and HDAC1 using chromatin immunoprecipitation followed by deep sequencing (ChIP-Seq) in human B-ALL cells (Nalm6 cell line). We identified 12 464 distinct binding sites for Ikaros and 9971 for HDAC1, and these were associated with 6722 and 6182 target genes, respectively (Figure 1a). Of these, 12% of the Ikaros-binding sites overlapped by at least 1 bp with 14.6% of the HDAC1-binding sites. The overlapping binding sites correlated with 934 gene targets (Figure 1a). ChIP-Seq data for Ikaros and HDAC1 were validated by quantitative chromatin immunoprecipitation (qChIP) analysis of the high- and low-rank ChIP-Seq peak values (Supplementary Figures S1 and S2). The peak distributions of Ikaros and of HDAC1 relative to target genes revealed that the binding of both proteins is highly enriched within ±3 kb from transcriptional start sites (Figure 1b).

We analyzed the effect of Ikaros and HDAC1 DNA binding on the surrounding chromatin. First, the genome-wide distribution of histone H3 trimethylation at lysine 4 (H3K4me^3^), lysine 27 (H3K27me^3^), lysine 36 (H3K36me^3^), or lysine 9 (H3K9me^3^), or acetylated at lysine 9 (H3K9ac) was determined by ChIP-Seq experiments in Nalm6 cells. ChIP-Seq data for histone modifications were validated by qChIP analysis of the high- and low-rank ChIP-Seq peak values (Supplementary Figures S3–S7). Next, we analyzed the distribution of chromatin modifications relative to (1) Ikaros peaks; (2) Ikaros–HDAC1 overlapped peaks; and (3) HDAC1 peaks. Most of the Ikaros and HDAC1 binding occurs within the promoters of target genes (Figure 1b). Thus, we compared the epigenetic changes that we observed in chromatin surrounding Ikaros, Ikaros–HDAC1 and HDAC1 peaks (Figures 1c–e), which are located within the promoter region, to epigenetic markers present in chromatin surrounding promoters across the genome, regardless of Ikaros and/or HDAC1 occupancy (Figure 1f).

We found that unique epigenetic changes are associated with Ikaros, Ikaros–HDAC1 and HDAC1 peaks. Ikaros peaks are associated with the presence of H3K4me^3^, H3K9me^3^ and H3K9ac histone modifications (Figure 1c). Ikaros–HDAC1 overlapped peaks correlated with a different chromatin environment that is characterized by the very strong presence of H3K4me^3^ and H3K27me^3^, moderate H3K9me^3^ and virtually absent H3K9ac (Figure 1d). HDAC1 peaks were also associated with the very strong presence of H3K27me^3^ and H3K4me^3^, and virtually absent H3K9ac. However, H3K9me^3^ was reduced as compared with Ikaros or Ikaros–HDAC1 peaks (Figure 1e). These results indicate that the binding of Ikaros, Ikaros–HDAC1 or HDAC1 is each associated with a distinct characteristic chromatin change that likely affects the expression of target genes. The specific distribution of histone modifications around Ikaros, Ikaros–HDAC1 or HDAC1 peaks were similar, regardless of whether these peaks were localized within promoter regions or other regions across the genome (Supplementary Figures S8–S10). Most of the specific epigenetic changes occur within 1 kb of the center of the Ikaros, Ikaros–HDAC1 or HDAC1 peaks. This suggests that binding of these proteins has a direct effect on chromatin remodeling and the observed epigenetic changes.

Our analysis demonstrates a strong association between HDAC1 occupancy and H3K27me^3^ (Supplementary Table S1). This is particularly pronounced at promoter regions—85% of all promoters with H3K27me^3^ showed HDAC1 binding (Figure 1g). This suggests that HDAC1 occupancy is the major determinant of the H3K27me^3^ marker. Further analysis demonstrates Ikaros–HDAC1 occupancy at 21% of all promoters with H3K27me^3^ in leukemia cells (Figure 1g). This suggests that Ikaros binding to promoters of its target genes can result in H3K27me^3^ via recruitment of HDAC1. These results show the importance of Ikaros' recruitment of HDAC1 in determining the global epigenetic signature in leukemia. We tested whether histone deacetylase activity is required for the formation of H3K27me^3^ in Nalm6 cells. Treatment of Nalm6 cells with the histone deacetylase inhibitor trichostatin resulted in strong reduction of H3K27me^3^ by western blot (Figure 1h), suggesting that histone deacetylase activity is essential for the presence of H3K27me^3^. These results demonstrate an essential role for histone deacetylase in the formation of H3K27me^3^ in B-ALL.

ChIP-Seq analysis of the epigenetic signature around Ikaros occupancy led to the hypothesis that DNA binding of Ikaros or Ikaros–HDAC1 complexes alters the transcription of their respective target genes by induction of distinct epigenetic changes. To test this hypothesis, we analyzed the effect of increased Ikaros expression on chromatin remodeling at promoters of genes that are regulated by Ikaros-only or by Ikaros–HDAC1 complexes. Recently, we reported that Ikaros represses the transcription of a large number of genes that promote cell cycle progression in leukemia.^15^ The epigenetic signatures at promoters of the cell cycle-promoting genes *CDC7* and *ANAPC7* (Ikaros-only targets), and *CDC2* and *ANAPC1* (Ikaros–HDAC1 targets) were compared in Nalm6 cells transduced with Ikaros or empty vector (control) using serial qChIP assays. Results showed that increased Ikaros expression is associated with unchanged H3K27me^3^, increased H3K9me^3^ and decreased H3K9ac in regulatory elements of the Ikaros-only targets, *CDC7* and *ANAPC7* (Figure 2a, Supplementary Figure S11a, red vs black lines). In contrast, in regulatory elements of the Ikaros–HDAC1 target genes *CDC2* and *ANAPC1,* increased Ikaros expression is associated with increased H3K27me^3^, unchanged H3K9me^3^ and decreased H3K9ac (Figure 2b, Supplementary Figure S11b, red vs black lines). These data identify specific epigenetic signatures induced by binding of Ikaros-only and Ikaros–HDAC1 complexes to promoters of Ikaros target genes in B-ALL.

Next, we studied how Ikaros loss-of-function or gain-of-function affects the transcriptional regulation and epigenetic signature of Ikaros target genes in primary high-risk B-ALL cells. In high-risk B-ALL, Ikaros function as a transcriptional regulator is severely impaired due to the deletion of one Ikaros allele and/or functional inactivation of Ikaros protein by Casein Kinase II (CK2) phosphorylation.^15^ Inhibition of CK2 has been shown to restore Ikaros activity as transcriptional regulator, resulting in transcriptional repression of Ikaros target genes that promote cell cycle progression.^15^ We analyzed the epigenetic signature at promoters of Ikaros and Ikaros–HDAC1 target genes in primary high-risk B-ALL (with loss of Ikaros function), and in primary high-risk B-ALL cells following treatment with CK2 inhibitors (with restored Ikaros function). In high-risk B-ALL, Ikaros DNA binding to the promoters of its target genes is impaired (Figures 2c and d, Supplementary Figures S12 and S13 black lines). Inhibition of CK2 with a specific CK2 inhibitor, CX-4945, restored Ikaros DNA binding to promoters and induced an epigenetic signature with high-level H3K9me^3^, reduced H3K9ac and the absence of H3K27me^3^ at the Ikaros-only target gene, *CDC7* (Figure 2c, Supplementary Figure S12a, red vs black lines). However, for the Ikaros–HDAC1 target, *CDC2*, restoration of Ikaros binding following CK2 inhibition results in a high level of H3K27me^3^, the loss of H3K9ac and largely unchanged H3K9me^3^ (Figure 2d, Supplementary Figure S12b, red vs black lines). Results obtained following the restoration of Ikaros function demonstrate that treatment of high-risk B-ALL cells with the CK2 inhibitor CX-4945 results in epigenetic changes that are remarkably similar to those found with increased Ikaros expression in Nalm6 (Figures 2c and d and Supplementary Figures S12 and S13c and d as compared with Figures 2a and b).

The distinct epigenetic changes that occur following the restoration of Ikaros binding to promoters of Ikaros-only and Ikaros–HDAC1 target genes were reproduced in cells derived from three different primary high-risk B-ALL following treatment with CK2 inhibitor CX-4945 (Figures 2c and d, Supplementary Figure S12). These results were also reproduced following treatment of high-risk primary B-ALL cells with a different CK2 inhibitor, TBB, (Supplementary Figures S13a and b compared with Figures 2a–d and Supplementary Figures S12 and S13c and d compared with Supplementary Figure 11).

In summary, our data reveal the mechanism by which chromatin remodeling and target gene expression are regulated by Ikaros alone and in complex with HDAC1 in B-ALL (Figure 2e). These data suggest that Ikaros can repress transcription of its target genes by inducing the formation of repressive chromatin via two distinct mechanisms: (1) direct Ikaros binding resulting in the formation of heterochromatin due to increased H3K9me^3^ and reduced H3K9ac; or (2) Ikaros recruitment of HDAC1, where the most prominent change is a strong increase in H3K27me^3^ along with reduced H3K9ac. In high-risk B-ALL, Ikaros ability to regulate chromatin remodeling of its target genes is impaired. In high-risk B-ALL with deletion of one Ikaros allele, inhibition of CK2 restores Ikaros-mediated epigenetic repression of the cell cycle-promoting genes. These data suggest that the ability to regulate chromatin remodeling is an essential part of Ikaros tumor-suppressor function. These studies provide new insight into the epigenetic regulation of gene expression in B-ALL and a rationale for the use of CK2 inhibitors as a novel treatment.

## Figures and Tables

**Figure 1:**
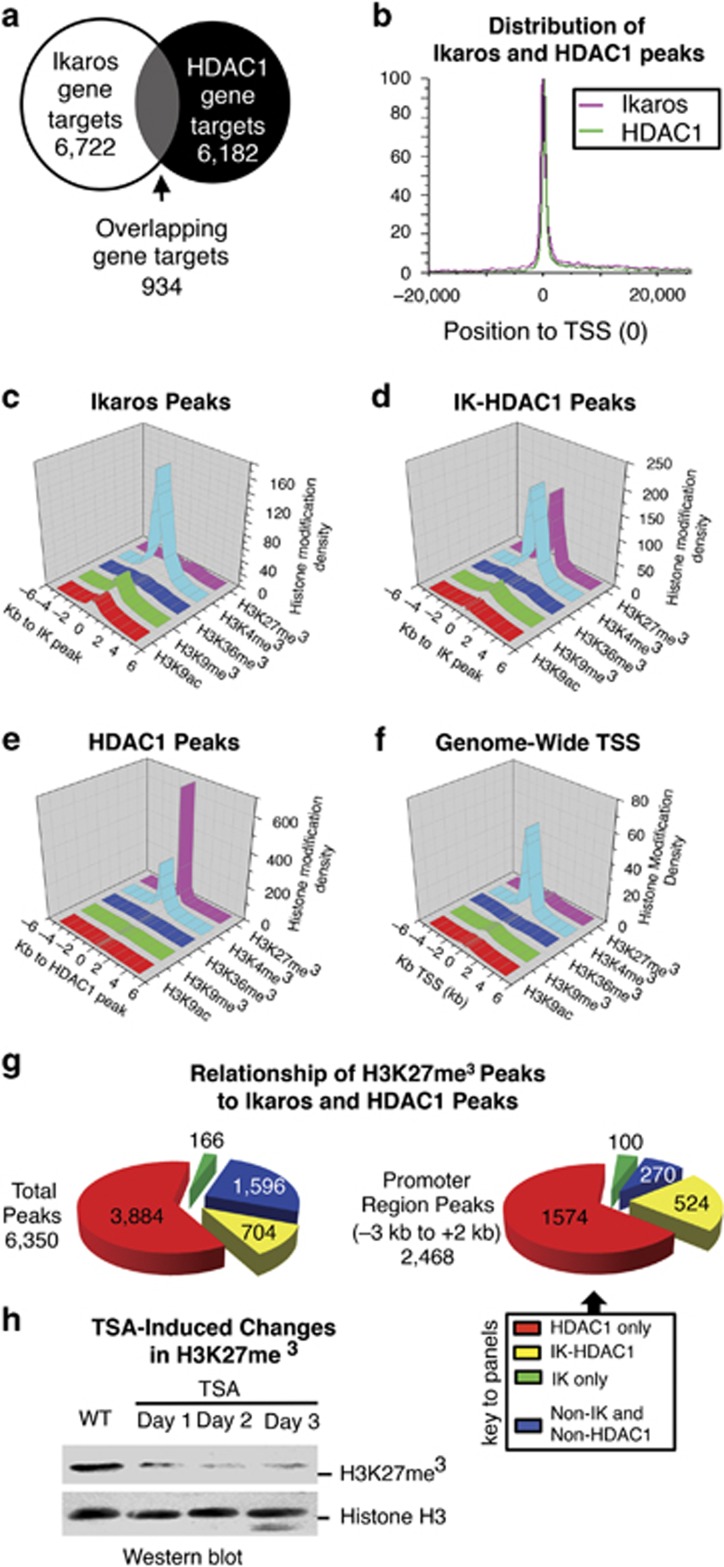
Genome-wide mapping of Ikaros and HDAC1 binding in B-ALL cells. (**a**) Ikaros and HDAC1 target genes identified by ChIP-Seq analysis of Nalm6 B-ALL cells. The overlapping gene targets have Ikaros and HDAC1 peaks overlapped by at least 1 bp. (**b**) The distribution of Ikaros and HDAC1 peaks around the transcriptional start sites (TSS). Peak numbers were normalized by treating the maximum possible peak number at a location as 100. (**c–f**) Specific epigenetic changes associated with Ikaros and HDAC1 occupancy. The distribution of histone modifications relative to the center of (**c**) the Ikaros peak; (**d**) the Ikaros peak in Ikaros (IK)-HDAC1 overlapped peaks; (**e**) the HDAC1 peak; (**f**) all TSS, genome wide. Graphed is the frequency of each particular epigenetic modifications per 100 Ikaros, or 100 Ikaros–HDAC1 peaks, over a 1 kb span. (**g**) Association of H3K27me^3^ with IK and HDAC1 occupancy, genome wide (left), or within the promoter region (right). Graphed are the number of H3K27me^3^ peaks located within 1 kb of IK, HDAC1, or IK–HDAC1 peaks, or outside of these regions (non-IK and non-HDAC1). (**h**) Effect of pan-HDAC inhibitor (TSA) on H3K27me^3^ level. Western blot of H3K27me^3^ in untreated Nalm6 cells and following TSA treatment at specific days are shown. The total level of histone H3 was used for normalization (bottom). WT, wild type.

**Figure 2:**
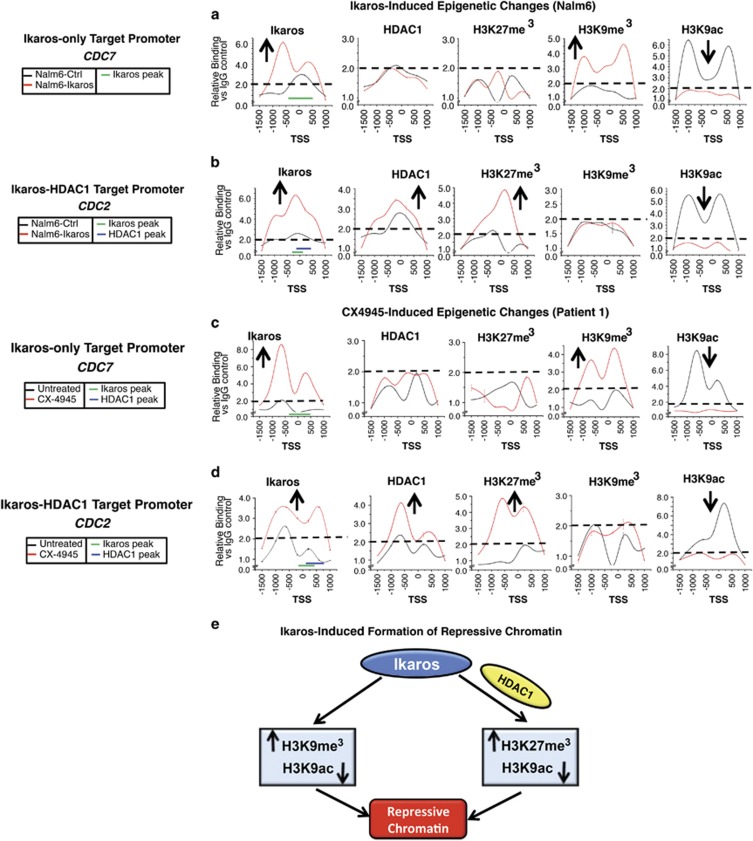
Ikaros-mediated chromatin changes in promoter regions of Ikaros target genes. (**a**, **b**) Epigenetic signature at promoters of Ikaros target genes following overexpression of Ikaros in Nalm6 cells (red line) or in control Nalm6 cells (black line). The binding of Ikaros and HDAC1, and the histone modification markers, H3K27me^3^, H3K9me^3^ and H3K9ac were detected by serial qChIP assays in a representative (**a**) Ikaros-only target gene (*CDC7*) and (**b**) IK–HDAC1 target gene (*CDC2)* in Nalm6 B-ALL cells. (**c**, **d**) Epigenetic signature at promoters of Ikaros target genes in primary high-risk B-ALL cells that carry deletion of one Ikaros allele (patient 1; black line) and following treatment with the CK2 inhibitor, CX-4945 (red line). The binding of Ikaros, HDAC1 and histone modification markers were detected by serial qChIP assays in the representative (**c**) Ikaros-only target gene (*CDC7*) and (**d**) IK–HDAC1 target gene (*CDC2*) in primary cells from patient 1. Patient characteristics are shown in Supplementary Table S2. Graphed data are means±s.d. of data obtained using five primer pairs that span the transcription start site (TSS) of indicated genes. In addition to the presented data, the serial qChIP assays for H3K4me^3^ did not show any changes following treatment with CX-4945 (data not shown). (**e**) Model of proposed epigenetic mechanisms for Ikaros- and IK–HDAC1-mediated regulation of gene expression.
