# Supplementary material for: Epigenetic regulation of gene expression by Ikaros, HDAC1 and Casein Kinase II in leukemia
Source: Leukemia. 2016 Jan 22;30(6):1436–40. doi: 10.1038/leu.2015.331 (PMC4889471; doi:10.1038/leu.2015.331)
Supplement: Supplementary Figures and Tables [file leu2015331x1.pdf]

Supplementary Figures and Tables

Epigenetic regulation of gene expression by Ikaros, HDAC1 and Casein Kinase II (CK2) in leukemia

Chunhua Song[1*], Xiaokang Pan[1*], Zheng Ge[2,1], Chandrika Gowda[1], Yali Ding[1], Hui Li[1], Zhanjun Li[3,1], Gregory Yochum[4], Markus Muschen[5], Qunhua Li[6], Kimberly J. Payne[7], and Sinisa Dovat[1,8]

[1]Pennsylvania State University Medical College, Department of Pediatrics, Hershey, 17033 PA
[2]The First Affiliated Hospital of Nanjing Medical University, Jiangsu Province Hospital, Department of Hematology, Nanjing 210029, China
[3]Jilin Province Animal Embryo Engineering Key Laboratory, College of Animal Science and Veterinary Medicine, Jilin University, Changchun, Jilin, 130062, China.
[4]Pennsylvania State University Medical College, Dept. of Biochemistry and Molecular Biology, Hershey, 17033 PA
[5]University of California San Francisco, San Francisco, CA
[6]Pennsylvania State University, Department of Statistics, University Park, PA
[7]Loma Linda University, Loma Linda, CA
[8]Corresponding Author

* These authors contributed equally to this work

qChIP Analysis of Ikaros Binding

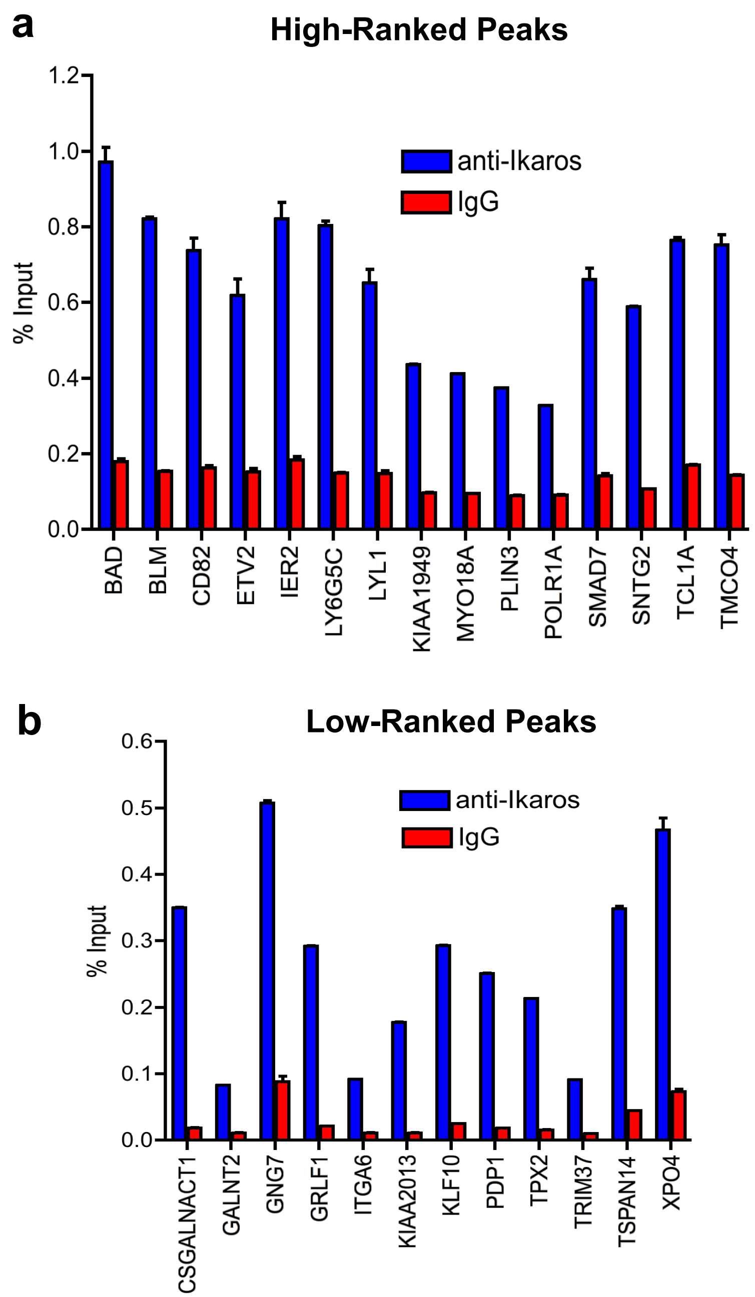

Supplementary Figure 1. Quantitative ChIP analysis of Ikaros binding in Nalm6 B-ALL cells. qChIP analysis of Ikaros occupancy at sites of (**a**) high- and (**b**) low-ranked ChIP-seq peak values. Primers for qChIP assays are shown in Supplementary Methods.

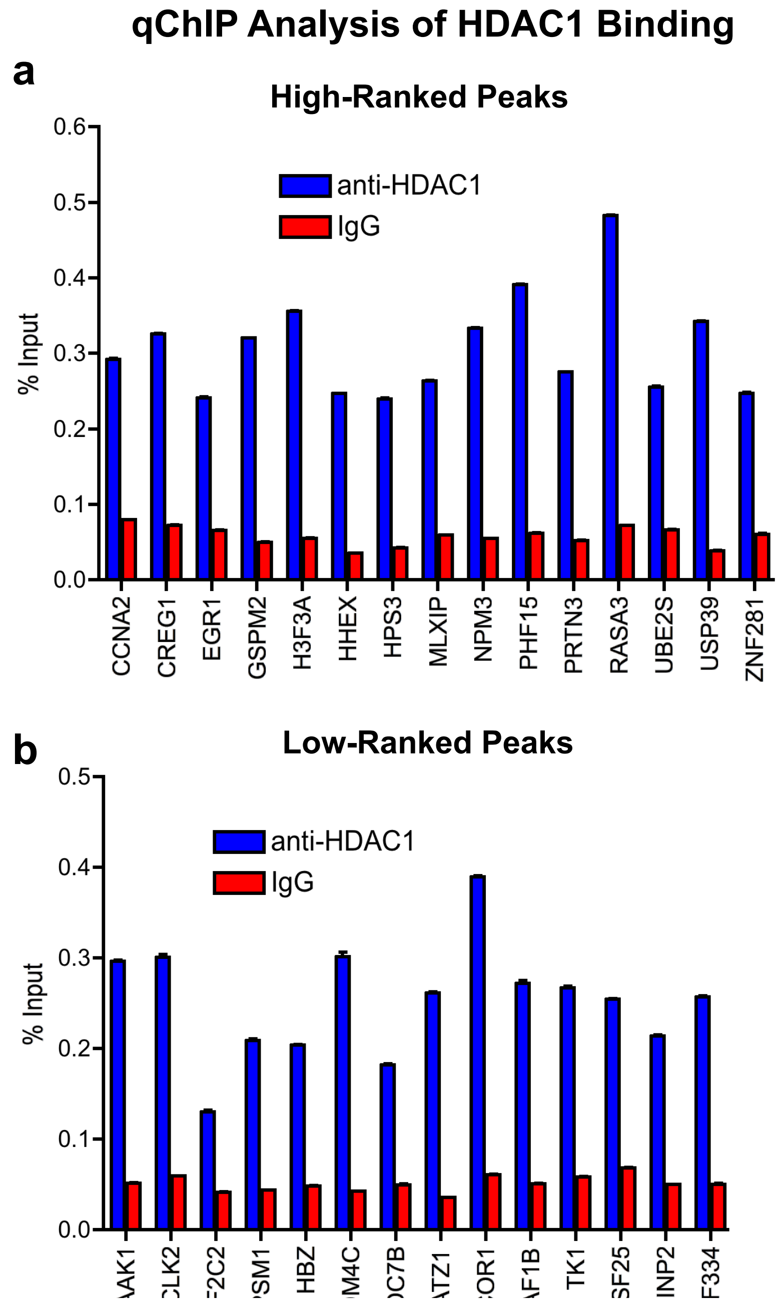

Supplementary Figure 2. Quantitative ChIP analysis of HDAC1 binding in Nalm6 B-ALL cells. qChIP analysis of HDAC1 occupancy at sites of (**a**) high- and (**b**) low-ranked ChIP-seq peak values. Primers for qChIP assays are shown in Supplementary Methods.

qChIP Analysis of H3K4me^3 Peaks

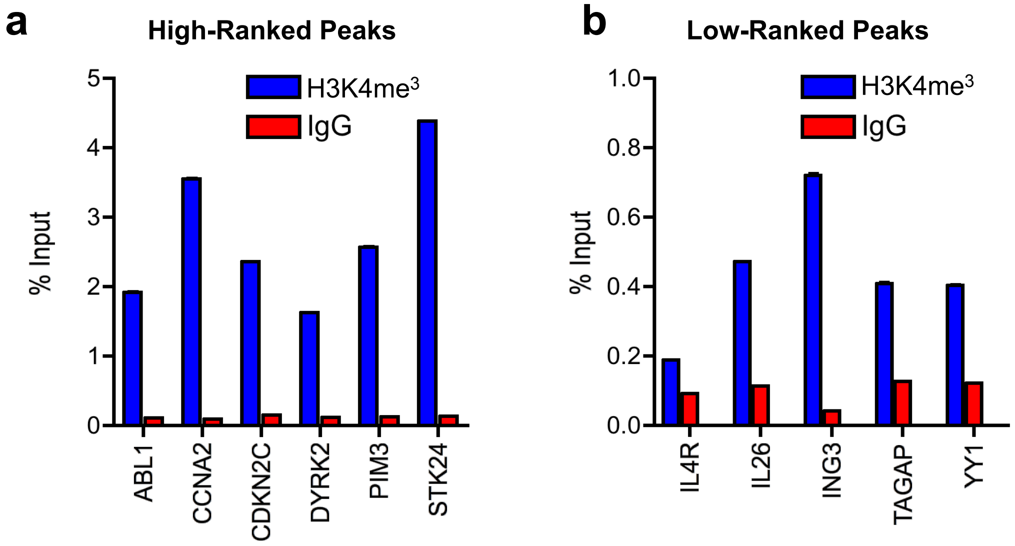

Supplementary Figure 3. Quantitative ChIP analysis of H3K4me^3 modifications in B-ALL cells. qChIP analysis of H3K4me^3 at sites of (**a**) high- and (**b**) low-ranked ChIP-seq peak values. Primers for qChIP assays are shown in Supplementary Methods.

qChIP Analysis of H3K27me^3 Peaks

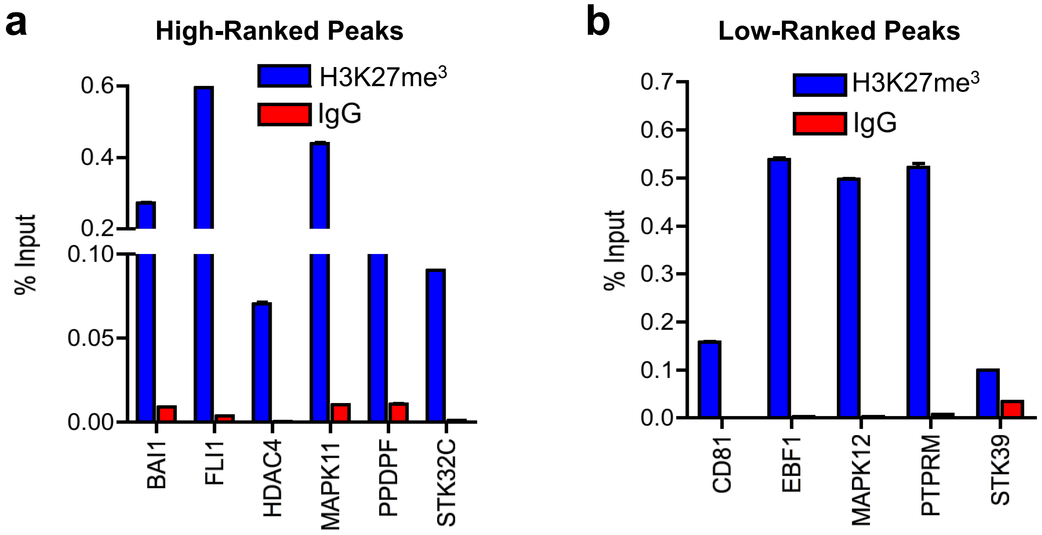

Supplementary Figure 4. Quantitative ChIP analysis of H3K27me^3 modifications in B-ALL cells. qChIP analysis of H3K27me^3 at sites of (**a**) high- and (**b**) low-ranked ChIP-seq peak values. Primers for qChIP assays are shown in Supplementary Methods.

qChIP Analysis of H3K36me^3 Peaks

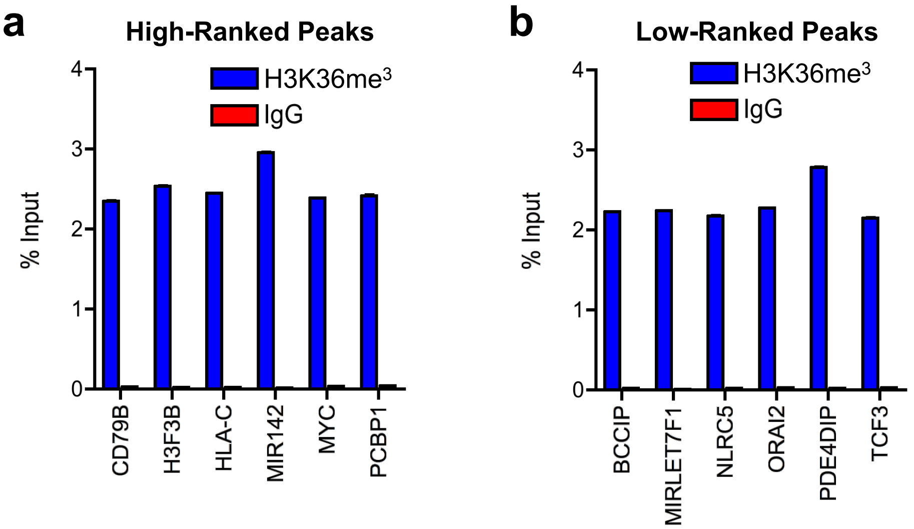

Supplementary Figure 5. Quantitative ChIP analysis of H3K36me^3 modifications in B-ALL cells. qChIP analysis of H3K36me^3 at sites of **a**, high- and **b**, low-ranked ChIP-seq peak values. Primers for qChIP assays are shown in Supplementary Methods.

qChIP Analysis of H3K9me^3 Peaks

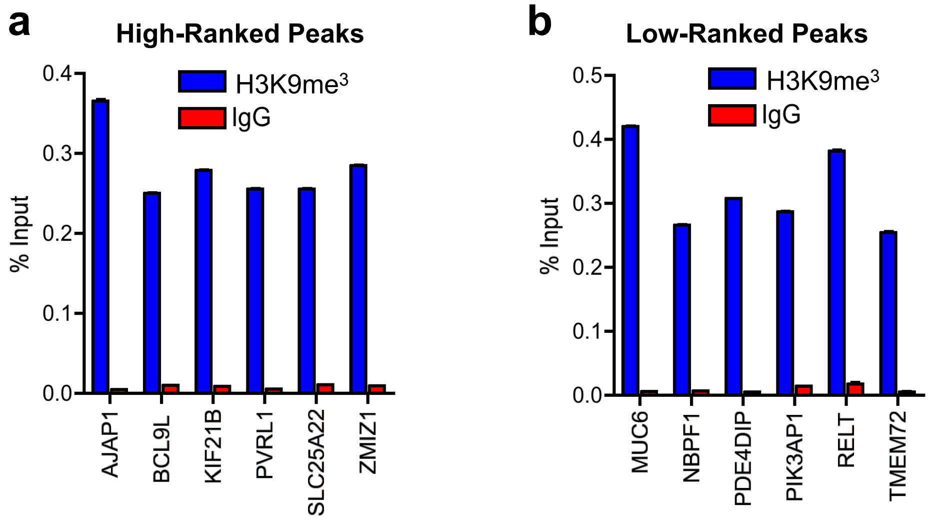

Supplementary Figure 6. Quantitative ChIP analysis of H3K9me^3 modifications in B-ALL cells. qChIP analysis of H3K9me^3 at sites of (**a**) high- and (**b**) low-ranked ChIP-seq peak values. Primers for qChIP assays are shown in Supplementary Methods.

qChIP Analysis of H3K9ac Peaks

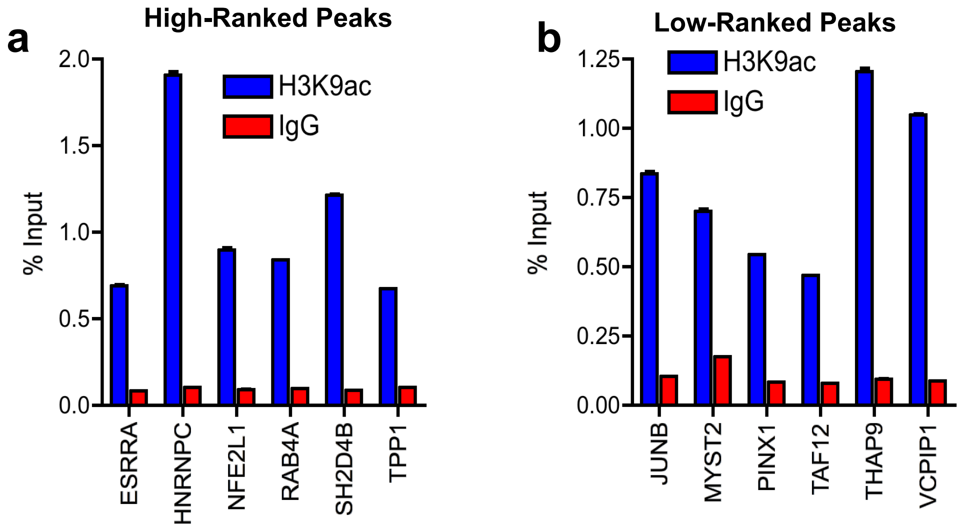

Supplementary Figure 7. Quantitative ChIP analysis of H3K9ac modifications in B-ALL cells. qChIP analysis of H3K9ac at sites of (**a**) high- and (**b**) low-ranked ChIP-seq peak values. Primers for qChIP assays are shown in Supplementary Methods.

Distribution of Histone Modifications Relative to Ikaros-Only Peaks

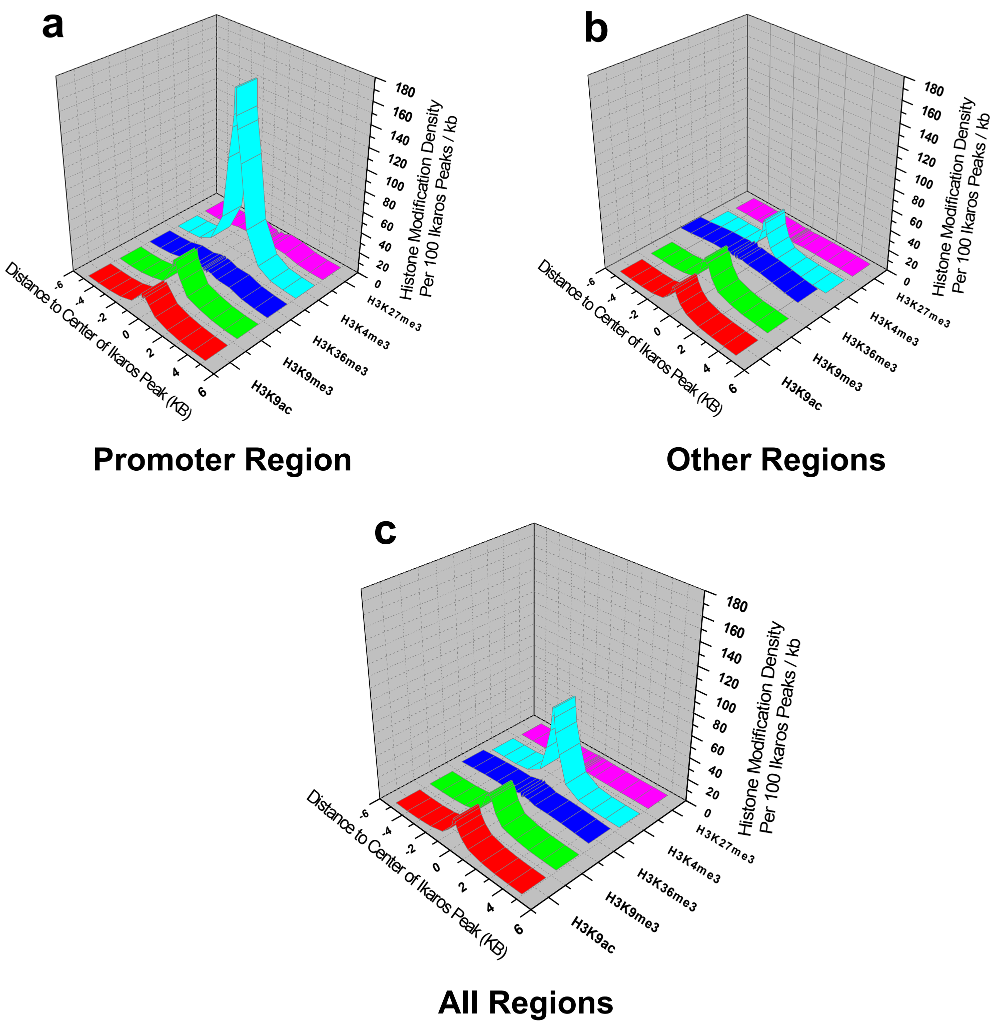

Supplementary Figure 8. Distribution of H3K9ac, H3K9me^3, H3K36me^3, H3K4me^3, and H3K27me^3 peaks relative to the center of the Ikaros peak. Distribution of histone modifications around Ikaros peaks that are located within (**a**) the promoter region, (**b**) other regions, or (c) all peaks, are shown.

Distribution of Histone Modifications Relative to Ikaros-HDAC1 Peaks

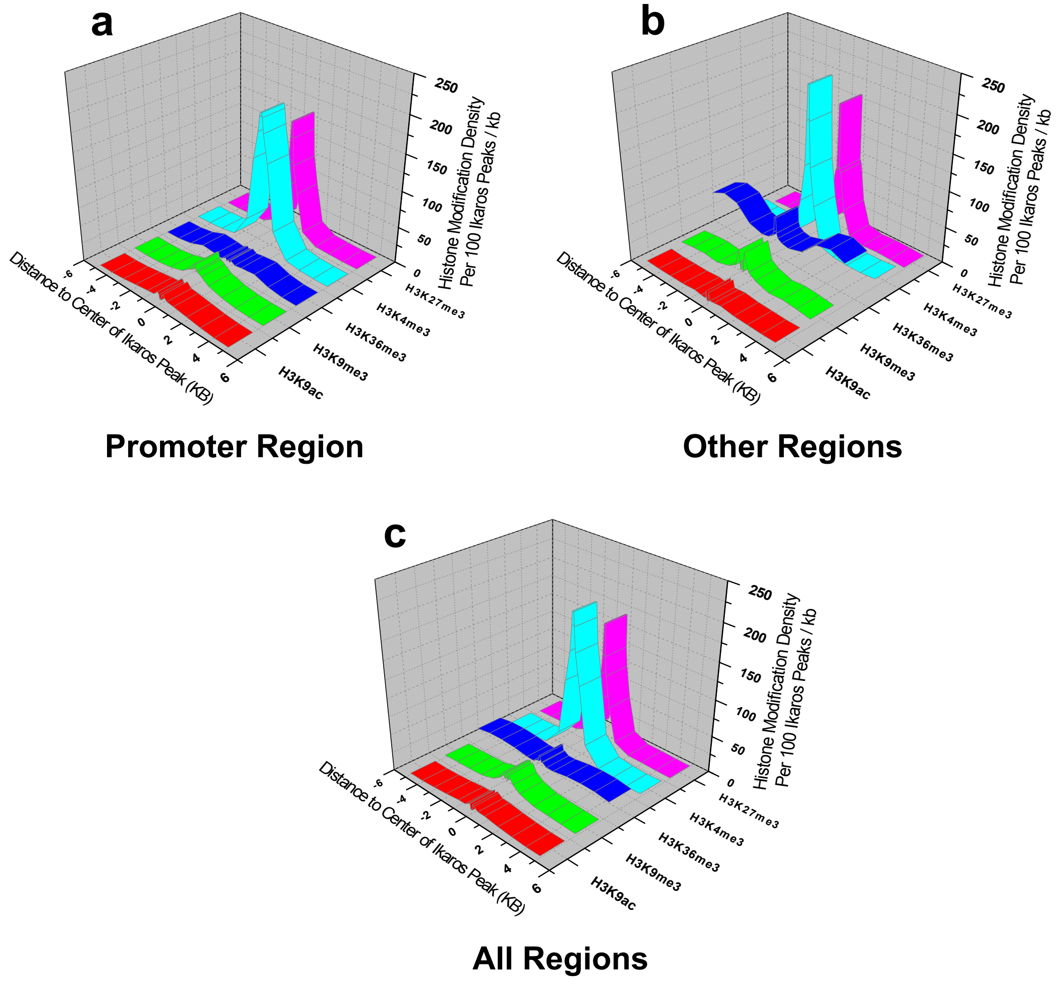

Supplementary Figure 9. Distribution of H3K9ac, H3K9me^3, H3K36me^3, H3K4me^3, and H3K27me^3 peaks relative to the center of the Ikaros-HDAC1 peak. Distribution of histone modifications around Ikaros-HDAC1 peaks that are located within (**a**) the promoter region, (**b**) other regions, or (**c**) all peaks, are shown.

Distribution of Histone Modifications Relative to HDAC1-Only Peaks

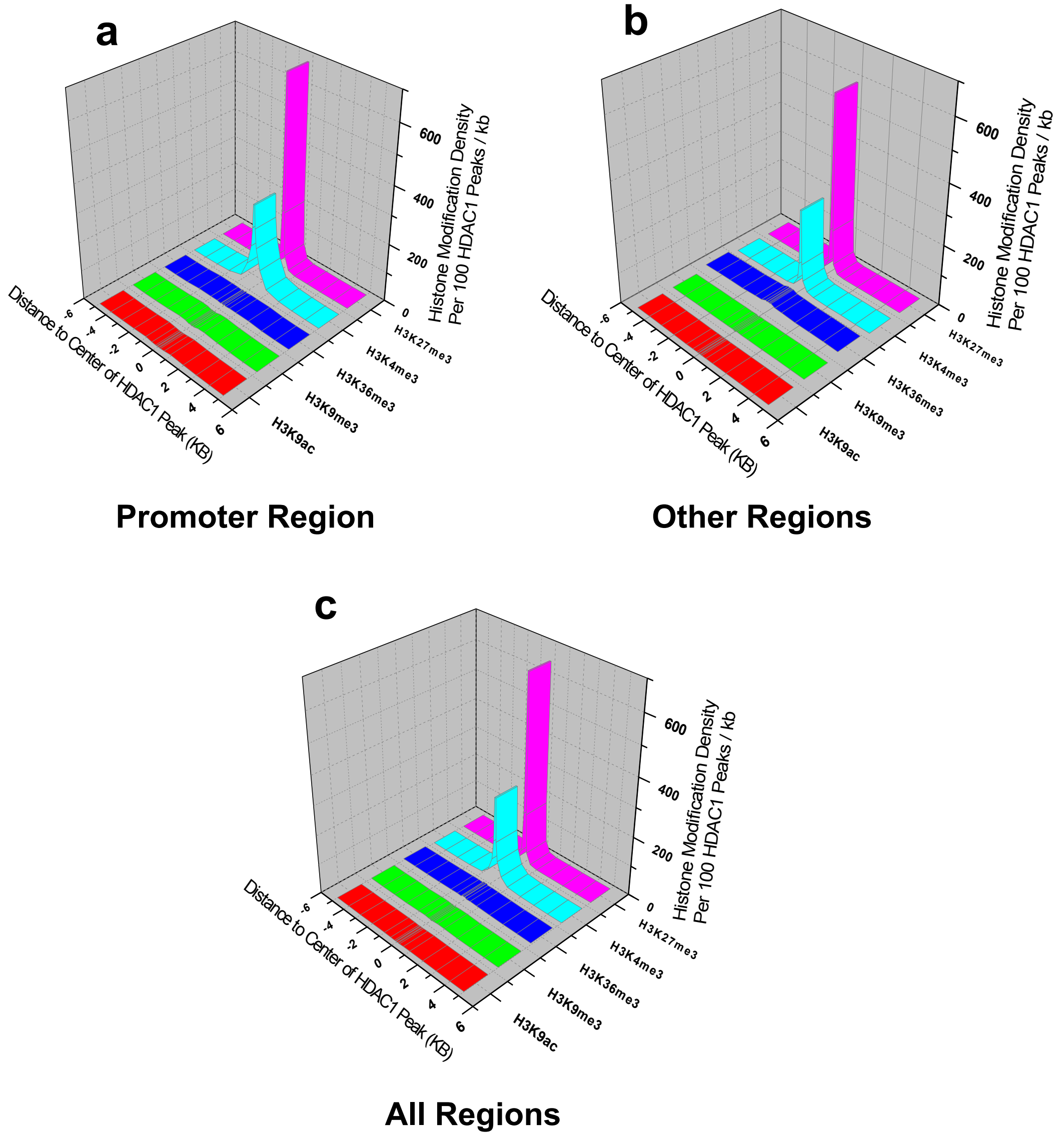

Supplementary Figure 10. Distribution of H3K9ac, H3K9me^3, H3K36me^3, H3K4me^3, and H3K27me^3 peaks relative to the center of the HDAC1 peak. Distribution of histone modifications around HDAC1 peaks that are located within (**a**) the promoter region, (**b**) other regions, or (**c**) all peaks are shown.

Ikaros-Induced Epigenetic Changes (Nalm6)

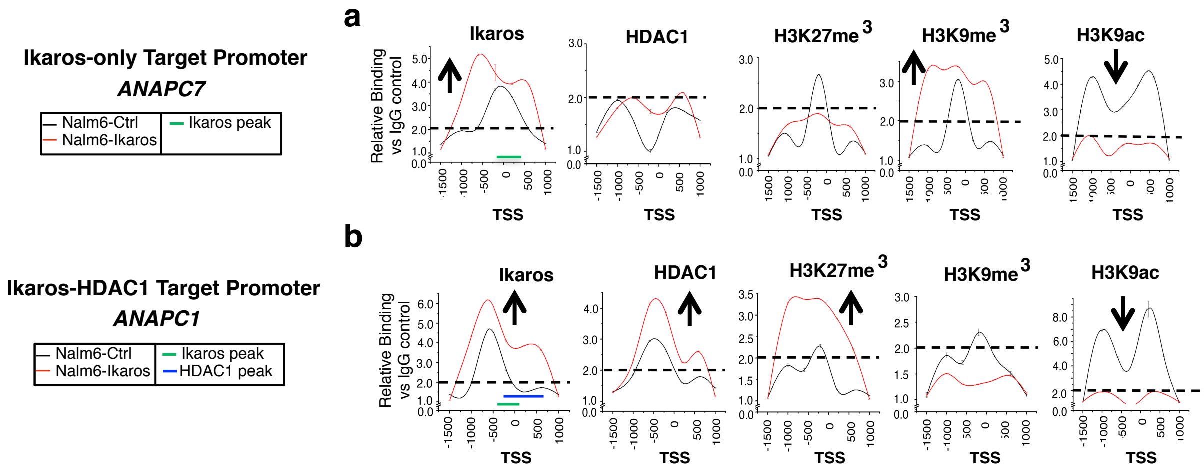

Supplementary Figure 11. Ikaros-mediated chromatin changes in promoter region of targets. The binding of Ikaros, HDAC1, and histone modification markers, H3K27me^3, H3K9me^3, and H3K9ac were detected by qChIP in the representative (**a**) Ikaros-only target gene (*ANAPC7*) and (**b**) IK-HDAC1 target gene (*ANAPC1*) in Nalm6 B-ALL cells with overexpressed Ikaros (red line) and in control Nalm6 cells (black line). Graphed data are means ± SD. Primers for qChIP assays are shown in Supplementary Methods. Green and Blue horizontal lines denote the presence of Ikaros and HDAC1 peaks, respectively, detected by ChIP-seq of Nalm6 cells.

CX4945-Induced Epigenetic Changes (Primary B-ALL)

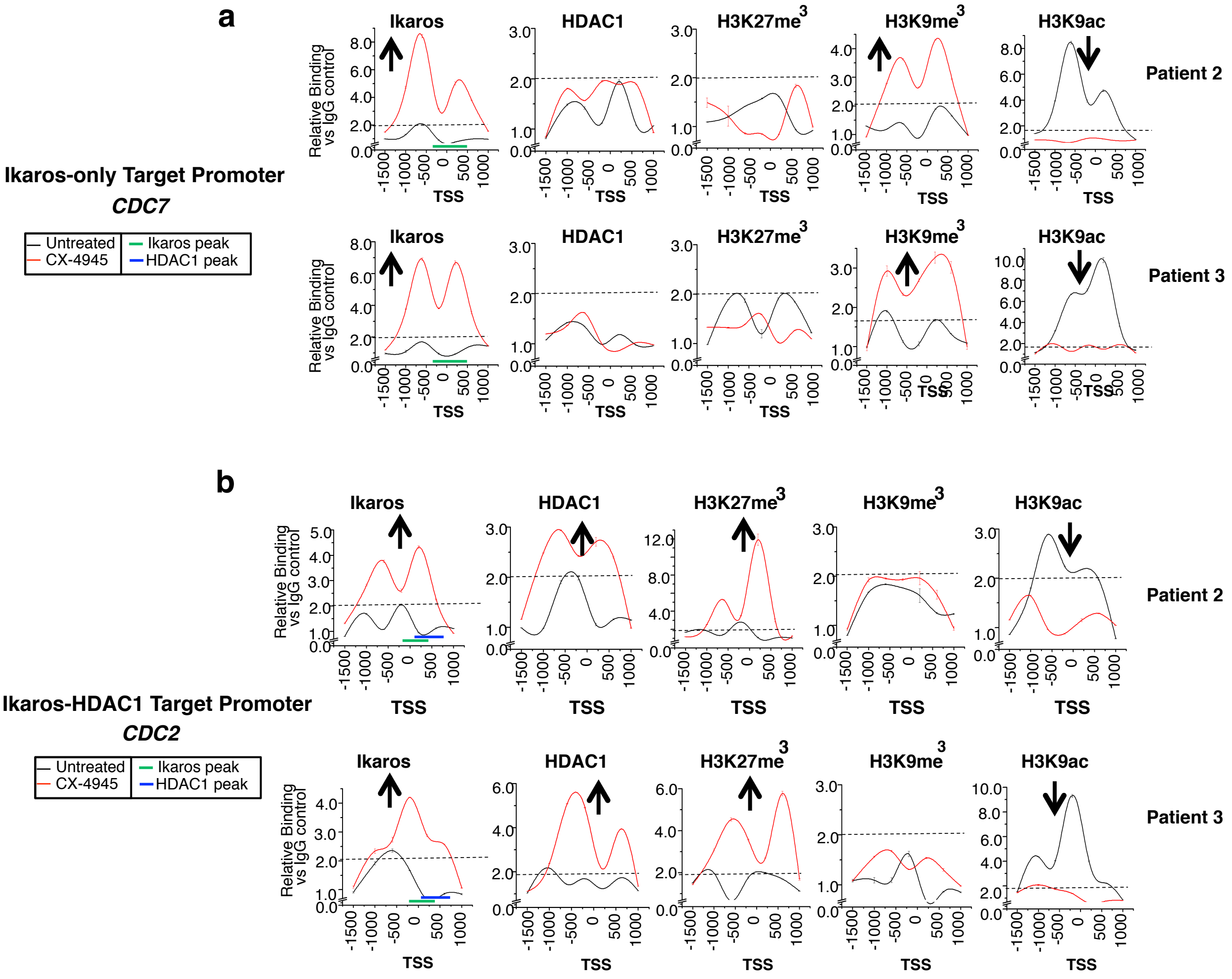

Supplementary Figure 12. Epigenetic changes at promoter regions of Ikaros target genes in primary cells of high-risk B-ALL following CK2 kinase inhibition with CX-4945. The binding of Ikaros, HDAC1, and histone modification markers H3K27me3, H3K9me3, and H3K9ac were detected by qChIP in a representative (**a**) Ikaros-only target gene (*CDC7*) and (**b**) IK-HDAC1 target gene (*CDC2*) in primary high-risk B-ALL cells that carry deletion of one Ikaros allele (patient 2 and patient 3). The qChIP results from untreated cells (black line) and following CK2 inhibition with CX-4945 (red line) are shown. Graphed data are means ± SD. Primers for qChIP assays are shown in Supplementary Methods. Green and Blue horizontal lines denote the presence of Ikaros and HDAC1 peaks, respectively, detected by ChIP-Seq of Nalm6 cells. In addition to presented data, the serial qChIP assays for H3K4me3 did not show any changes following treatment with CX-4945 (data not shown).

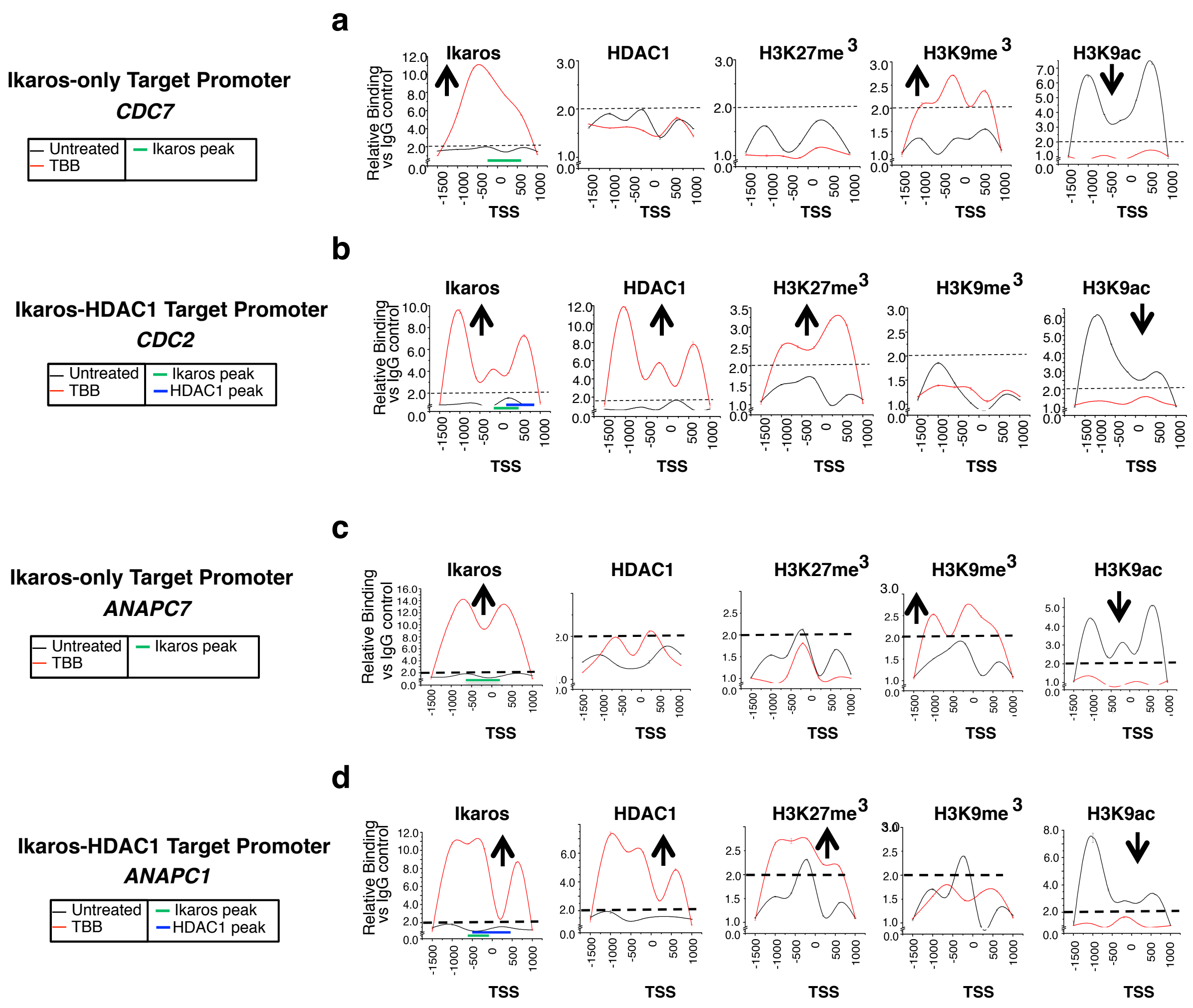

Supplementary Figure 13. Epigenetic changes at promoter regions of Ikaros target genes in primary cells of high-risk B-ALL following CK2 kinase inhibition with TBB. The binding of Ikaros, HDAC1, and histone modification markers, H3K27me^3, H3K9me^3, and H3K9ac were detected by qChIP in representative (**a, c**) Ikaros-only target genes (*CDC7*, *ANAPC7*) and (**b, d**) IK-HDAC1 target genes (*CDC2, ANAPC1*) in primary high-risk B-ALL cells that carry deletion of one Ikaros allele (patient 1). The qChIP results from untreated cells (black line) and following CK2 inhibition with TBB (red line) are shown. Graphed data are means $\pm$ SD. Primers for qChIP assays are shown in Supplementary Methods. Green and blue horizontal lines denote the presence of Ikaros and HDAC1 peaks, respectively, detected by ChIP-seq of Nalm6 cells. In addition to presented data, the serial qChIP assays for H3K4me^3 did not show any changes following treatment with TBB (data not shown).

Supplementary Table 1: Permutation Test of Histone Modification Peaks Overlapped by HDAC1 Peaks.

Histone Modification	Overlapped Peak Numbers	Average Permuted Peak Numbers	P-value
H3K27me^3	3884	48	$< 2.2e^{-16}$

We observed that 3884 of 6351 H3K27me^3 peaks were overlapped by 8512 HDAC1 peaks without Ikaros peaks overlap by at least 1 bp (Fig. 2g). To assess significance, we generated 6351 H3K27me^3 peaks and 8512 HDAC1 peaks of the same width as those observed, randomized their genomic location within the same chromosomes as those observed, and calculated overlaps between H3K27me^3 and HDAC1 peaks. This permutation was performed 1000 times. The distributions of 1000 permuted peak numbers were compared to a Gaussian distribution using a Q-Q plot and found to be normal in R. P-values were then calculated *via* one sample T-test with the observed overlaps compared with the distribution generated using permuted peaks. The P-value was $< 2.2e^{-16}$.

Supplementary Table 2: Patient Characteristics

Patient Designation	Gender	Age	Genetic Abnormalities	High-Risk B-ALL Features
Patient 1	M	10 years	*Ikaros* deletion	> 1 x10^6 cells/ml in peripheral blood; 99% blasts; *Ikaros* deletion; age at diagnosis; Died soon after diagnosis
Patient 2	M	18 years	*Ikaros* deletion	>8x10^5 cells/ml in Periphery blood; 97% blasts; *Ikaros* deletion; 18 years of age at diagnosis; CRLF2-high
Patient 3	M	13 years	*Ikaros* deletion<br>*PAX5* deletion<br>*BCR-ABL*	*Ikaros* deletion: *BCR-ABL*
