# Supplementary material for: Epigenetic regulation of gene expression by Ikaros, HDAC1 and Casein Kinase II in leukemia
Source: Leukemia. 2016 Jan 22;30(6):1436–40. doi: 10.1038/leu.2015.331 (PMC4889471; doi:10.1038/leu.2015.331)
Supplement: Supplementary Methods [file leu2015331x2.pdf]

Supplementary Methods

Epigenetic regulation of gene expression by Ikaros, HDAC1 and Casein Kinase II (CK2) in leukemia

Chunhua Song[1*], Xiaokang Pan[1*], Zheng Ge[2,1], Chandrika Gowda[1], Yali Ding[1], Hui Li[1], Zhanjun Li[3,1], Gregory Yochum[4], Markus Muschen[5], Qunhua Li[6], Kimberly J. Payne[7], and Sinisa Dovat[1,8]

[1]Pennsylvania State University Medical College, Department of Pediatrics, Hershey, 17033 PA
[2]The First Affiliated Hospital of Nanjing Medical University, Jiangsu Province Hospital, Department of Hematology, Nanjing 210029, China
[3]Jilin Province Animal Embryo Engineering Key Laboratory, College of Animal Science and Veterinary Medicine, Jilin University, Changchun, Jilin, 130062, China.
[4]Pennsylvania State University Medical College, Dept. of Biochemistry and Molecular Biology, Hershey, 17033 PA
[5]University of California San Francisco, San Francisco, CA
[6]Pennsylvania State University, Department of Statistics, University Park, PA
[7]Loma Linda University, Loma Linda, CA
[8]Corresponding Author

* These authors contributed equally to this work

Cell Culture and Reagents

CX-4945 was a gift from Cylene Pharmaceuticals (San Diego, CA). 4,5,6,7-Tetrabromobenzotriazole (TBB) was purchased from Sigma (St. Louis, MO). The Nalm6 cell line was obtained from American Type Culture Collection (ATCC; Rockville, MD) and was grown in RPMI 1640 (Mediatech, Manassas VA) with 10% heat-inactivated Fetal bovine serum (FBS) (HyClone, Rockford, IL), 1% penicillin-streptomycin and 1% L-glutamine until exponential growth was obtained.

Patient Samples

Anonymous patient samples were provided from the USC Norris Comprehensive Cancer Center, Los Angeles, CA and Loma Linda University, Loma Linda, CA under protocols approved by the Institutional Review Board of the University of Southern California Health Sciences Campus and the Institutional Review Board of Loma Linda University. The wild type full-length *Ikaros* or *Ikaros* deletion in the patients' samples were originally confirmed by western blot[1] and/or genomic sequencing. Patient characteristics are shown in Supplementary Table 2. For TBB or CX-4945 treatment, the primary pre-B ALL cells were briefly (1-2 days) grown in the same conditions as the Nalm6 cell line.

Plasmid Construction, Retroviral Gene Transfer and Cell Sorting

Wild-type human HA-tagged Ikaros (*IKZF1*) cDNA was cloned by BglII and EcoRI site into the pMSCV bicistronic retroviral vector (MIG vector) which contains a 5' long-terminal-repeat-driven *Ikaros*, internal ribosome entry site (IRES), and enhanced green fluorescent protein (*EGFP*).[2-4] Retroviruses were produced by transient transfection in amphotropic packaging 293 cell lines as described previously.[5] Nalm6 cells were plated in 24-well platea at $4x10^5$ cells/well and suspended in retroviral supernatants with 12.5 mg/ml polybrene and centrifuged 1,400 *g*, at 32 °C, for 1h. The cells were then suspended in fresh 10% FBS RPMI 1640 and cultured at 37°C, 5% CO2 incubator for 3 days. The cells are Ficolled and the GFP(+) cells are sorted with FACSAria High speed sorter (Becton Dickinson). The sorted cells are cultured for further RNA isolation and ChIP assay.

ChIP-Seq Assays

ChIP-Seq analyses for Ikaros, HDAC1, and histone modification markers (histone H3K9ac, $H3K9me^3$, $H3K27me^3$, $H3K4me^3$ and $H3K36me^3$) were performed as previously described.[6, 7] Nalm6 cells ($3x10^8$ for Ikaros, $1x10^8$ for HDAC1, and $5x10^7$ for epigenetic markers) were cross-linked for 10 min by adding 1% formaldehyde to the growth media. Chromatin was fragmented using the Bioruptor sonicator (Diagenode) for 30 min (30s pulses, 30s pauses in between) to produce fragments ~400nt in size. ChIP assays were performed as previous reported.[6, 7] The affinity purified anti-Ikaros antibody,[1] anti-HDAC1 (Abcam, ab7028) and anti-histone modification markers antibodies (H3K9ac-Abcam, ab4441;$H3K9me^3$-Abcam, ab8898; $H3K27me^3$-Millipore, 07-449; $H3K4me^3$-Abcam, ab8580; $H3K36me^3$-Abcam, ab9050) pre-coated onto Goat-anti-rabbit IgG Dyneabeads (Invitrogen) were used and incubated with chromatin overnight at cold room.

Protein/DNA complexes were captured with a Magnetic Particle Concentrator (Invitrogen). Crosslinks were reversed. Samples were treated with proteinase K and RNase A. DNA was then recovered using the QIAquick PCR Purification kit (QIAGEN). ChIP libraries were created using 18 cycles amplification with ChIP-seq DNA sample prep kit (Illumina), in which libraries were run on a 2% agarose gel, and the 200-400 bp fraction was extracted and purified using DNA gel extraction kit (QIAGEN). The resulting library was validated by using Agilent Technologies 2100 Bioanalyzer.

Detection of ChIP-Seq Peaks

ChIP-Seq sequences were generated by Illumina Hiseq 2000 or Genome Analyzer II and then were mapped onto HG19 (Huaman Genome version 19 from NCBI) using ELAND algorithm[8] at the Genome Sequencing Center, University of Washington, Seattle, WA. Raw ChIP-Seq data are available on the NIH GEO database ChIP-seq data are accessible on GEO with an access number of GSE44218 at the link:

http://www.ncbi.nlm.nih.gov/geo/query/acc.cgi?acc=GSE44218.

CisGenome 2.0 (ref 9) was used to detect binding peaks for the Ikaros transcription factor, the HDAC1 enzyme, and the histone modification markers H3K4me^3 and H3K27me^3 on HG19 (Human Genome version 19 from NCBI). Parameters lpcut = 3 and c = 2 were selected for Ikaros, lpcut = 3 and w = 5 for HDAC1, and w = 5 for H3K4me^3 and H3K27me^3 datasets, respectively. Other parameters remain in default to run the program. Peaks with FDR ≤ 0.05 and Log2FC ≥ 1 were selected for further analysis. SISSRS (ref 10) was applied to find peaks for the histone modification markers H3K9ac, H3K9me^3 and H3K36me^3 with parameter P-value ≤ 0.05 and other parameters remain in default. The output peaks with FC ≥ 2 were kept for further data analysis.

Annotation of ChIP-Seq Peaks

Peak annotation with associated genes was retrieved using Perl script. In this script program, the peak information was inputted from the CisGenome output peak files in “COD” format. Then, the nearest gene of each peak and associated locations (gene start, gene end, 5’UTR, 3’UTR, exon and intron) on the human genome was identified using the CisGenome built-in program “refgene_getnearestgene” and “refgene_getlocationsummary” with parameters r = 0 (TSS-up, TES-down), up=100000 (BP) and down=100000 (BP), respectively. The gene names from HGNC (ref 11) and expression levels from microarray data analysis[12] were also inputted and passed. The output data file includes the integrated information of peaks, genes and gene expression levels.

Distribution of ChIP-Seq Peaks in Different Gene Regulatory Regions

The position of peaks in relation to TSS (Transcriptional Start Site) and other sites was determined and counted by a Perl script program. In this program, the maximum number of peaks at a position was also recorded. The percentage of the number of peaks spanning each position over the maximum number of peaks at a position was computed and then outputted into a tab-delimited text file. Finally, the output data was plotted in Microsoft Excel (Figure 1b). For Figure 1g, the number of peaks in the promoter and the total peaks were calculated using Perl script and then the distribution of the peaks was depicted in a pie chart using Microsoft Excel.

Determining Relationship of Ikaros ChIP-Seq Peaks to ChIP-Seq Peaks for HDAC1 and Histone Modification Markers

Ikaros peaks being overlapped by HDAC1 peaks were studied by looking into the different distances, 100BP, 500BP, 511BP (at least 1BP overlapped), 1KB, 3KB, 5KB, 10KB, 20KB, 50KB and 100KB from the center of Ikaros peaks to the center of HDAC1 peaks. Then, the peaks of histone modification (HM) markers H3K9ac, H3K9me^3, H3K36me^3, H3K4me^3 and H3K27me^3 were mapped onto the Ikaros-HDAC1 overlapped peaks in different distances of 50BP, 100BP, 200BP, 500BP, 1KB, 2KB, 3KBP, 4KB and 5KB from the centers of the HM peaks to the centers of Ikaros within Ikaros-HDAC1 overlapped peaks with a Perl script program. Similarly, the overlapping between HM peaks and Ikaros without HDAC1 and that between HM peaks and HDAC1 peaks without Ikaros were achieved.

The histone modification densities per 100 Ikaros peaks (without HDAC1 overlapping within 20KB)/KB, per 100 HDAC1 peaks (without Ikaros overlapping within 20KB)/KB and per 100 IK-HDAC1 peaks (with at least 1BP overlapping)/KB were calculated and plotted into 3D graphs using Original software package.

Validation of ChIP-Seq by Quantitative ChIP-qPCR (qChIP)

The low-rank and high-rank peaks for each ChIP-seq for Ikaros and HDAC1 as well as histone modifications were validated by qChIP. qChIP data for Ikaros and HDAC1 are shown in **Supplementary Figure 1-2**. qChIP data for histone modifications are shown in **Supplementary Figures 3-7**.

For qChIP assays, cells were collected by centrifugation at 2000g for 10 min and cross-linked in cross-link solution containing 1% formaldehyde for 10 min on ice. The reaction was stopped by adding glycine to a final concentration of 0.125 M. After 5 min, cells were washed twice with ice-cold PBS and centrifuged at 1,500-2,000 *x* g for 5 min at 4°C. Supernatant was discarded, and cell pellets were flash frozen in liquid nitrogen and stored at -80°C. Ikaros ChIP samples were

prepared as follows: Nalm6 cells ($2x10^7$ cells) or primary leukemia cells (4-$10x10^6$) per condition was treated with solution I (50mM Hepes KOH, pH7.5, 140mM NaCl, 1mM EDTA, 10% Glycerol, 0.5% NP-40, 0.25% Triton X-100, protease inhibitor) with rotation at 4°C for to 10min, after centrifugation at 3000RMP for 10min. The resulting pellet was treated with solution II (0.2M NaCl, 1mM EDTA pH8.0, 0.5mM EGTA pH8.0, 10 mM Tris pH8.0, protease inhibitor) with rotation for 10min at room temperature. The chromatin was fragmented in solution III (1mM EDTA pH8.0, 0.5mMEGTA pH8.0, 10mM Tris pH8.0, protease inhibitor) with a Bioruptor (Diagenode, Denville, NJ) for 14 min (30 s pulses, 90 s pauses) to obtain an average size of 400 bp and the chromatin was centrifuged at 4000RPM for 10min at at 4°C and 10% glycerol was added.

ChIP assays were performed by incubation of the chromatin in the buffer (1% triton X-100, 0.1% deoxycholate, 1x TE and protease inhibitor) with 20mg affinity-purified rabbit polyclonal anti-Ikaros antibody or normal rabbit IgG (Abcam, ab46540) as a control which was pre-coated onto Goat-anti-rabbit IgG Dyneabeads (Invitrogen). Following overnight incubation at 4°C, protein/DNA complexes were captured with a Magnetic Particle Concentrator (Invitrogen). Beads were washed with 1ml/time RIPA buffer (50mM Hepes pH8.0, 1mM EDTA pH8.0, 1% NP-40, 0.7deoxycholate, 0.5M LiCl) for 8 times and 1ml TE. After removal of TE, DNA was eluted with 50 μl elution buffer (10mM Tris pH8.0, 1mMEDTA, 1%SDS). Crosslinks were reversed in the presence of 0.6 M NaCl at 65°C overnight. Samples were treated with proteinase K Mix (14ug protease K and 3ug glycogen in 1xTE) for 2hrs at 37°C, extracted with phenol/chloroform, and then treated with 10 μg RNaseA for 2hrs at 37°C, and DNA was recovered using the QIAquick PCR Purification kit (QIAGEN).

Enrichment of the ChIP sample over input was evaluated by qPCR with specific primers (see below) in the promoter region of target genes. Three or more technical replicates were performed for most sites. Fold enrichment is calculated by the formula: $2^{-CTsample}/2^{-CTinput}$, where CT represents threshold cycle number of sample and input. Relative concentration of ChIP-qPCR product was presented as the fold change of DNA-Ikaros complex level in samples relative to IgG control. qChIP assays for HDAC1 and histone modification markers were performed with the same protocol as Ikaros qChIP except using $1x10^7$cells. Primers for qChIP are as follows:

Primers for qChIP Validation of Ikaros High-Ranked Peaks (Supplementary Figure 1a)

BAD-F	5'- AAAGGATCCTCCCGCTTCA-3'	BAD-R	5'- CGATGGTGAGCGCCTGTAAT-3'
BLM-F	5'- TGATGTTGGCCTTTTTTATGCA-3'	BLM-R	5'- CGTGGATTTGGGCAAGTTACA-3'
CD82-F	5'- AACCACTTCCTGGCCTGAGA-3'	CD82-R	5'- GGCATAACCCCGCCCTAT-3'
ETV2-F	5'- AGTCGGACCGTGCCAGTTT-3'	ETV2-R	5'- AGTCTTTGCGGCCCTCTCA-3'
IER2-F	5'-GGACAGGCTGAAGAACAATGG-3'	IER2-R	5'-GCTGCACCCCGAGGAAA-3'
LY6G5C-F	5'- GACCGGACTCGGGAATAGC-3'	LY6G5C-R	5'- CACATTGCATTTGGGTCAAGA-3'

LYL1-F	5'-ACCGGAAGCTGAGCAAGAAC-3'	LYL1-R	5'-CCAGGAAGCCGATGTACTTCA-3'
KIAA1949-F	5'- CCCTCCCTCCCAAGAACTG-3'	KIAA1949-R	5'- CCAGGATGGCAGGAAAAGAG-3'
MYO18A-F	5'- GCCCAAGCACCGCATCT-3'	MYO18A-R	5'- GAGCCCAGGTGTCAGGAAAA-3'
PLIN3-F	5'- GCGCCCCCAGTTTTCAC-3'	PLIN3-R	5'- GCTGTTGTCCTGGCAACCA-3'
POLR1A-F	5'- TGGGACGGGAGGCTTCTC-3'	POLR1A-R	5'- GCTCACCTGAATGTAGCAACAGA-3'
SMAD7-F	5'- GGGTTCCCTTCCAGGTTTTC-3'	SMAD7-R	5'- CAGGAGCAGTGCCCGTAGAC-3'
SNTG2-F	5'- GGATGAGGAGGAGAGGGAGAA-3'	SNTG2-R	5'- TGCAGAAATGCTCCCGTAATG-3'
TCL1A-F	5'- TTGTAGGCAGCCCCCTTTATC-3'	TCL1A-R	5'- GCCAGCTGAAACTTGTGAAGTACTT-3'
TMCO4-F	5'- GCAGTGGTGCGATGTCAGTT-3'	TMCO4-R	5'- AGGCACAAGAGTCGCTTGAAC-3'

Primers for qChIP Validation of Ikaros Low-Ranked Peaks (Supplementary Figure 1b)

CSGALNACT1-F	5'- CGCTGCCAAGCGTGTTG-3'	CSGALNACT1-R	5'- CCACCTGGGCCACATCTG-3'
GALNT2-F	5'- AAGCCGTGCCTTGTTGGAT-3'	GALNT2-R	5'- CCCACCCAGCACATCACAT-3'
GNG7-F	5'- CCTGGCCAACATAGTGAAACC-3'	GNG7-R	5'- CACGCCCGGCTAATTTTG-3'
GRLF1-F	5'- TGCTGGAAGCCGGGAAT-3'	GRLF1-R	5'- TCTAGATGGCGCCAAGTCAA-3'
ITGA6-F	5'- CATATAAGACATGTGGGTGTGTGGTA-3'	ITGA6-R	5'- CACTCCAAATGCACCCTGTATC-3'
KIAA2013-F	5'- CAGAGGAAATTGTGGGTACTCTTGT-3'	KIAA2013-R	5'- CTGTACTGCGCTGCCAACAT-3'
KLF10-F	5'- TGCCCGGGATCCTCCTA-3'	KLF10-R	5'- TGCTCCAAGGAGATGTTTAAGCT-3'
PDP1-F	5'- CCCTTCAGGGTGAGAAACAGAT-3'	PDP1-R	5'- GAGTTCCACACCGCTGCAA-3'
TPX2-F	5'- CGGGAGCCTGGAATTGCT-3'	TPX2-R	5'- CAGCCTAGTCGAATGCACCAA-3'
TRIM37-F	5'- CAGAGAGAACCACTAACCATGAACCT-3'	TRIM37-R	5'- TGTGGCCCGCACTTTGA-3'
TSPAN14-F	5'- CCCCACTGGGTTCCTGGTA-3'	TSPAN14-R	5'- TGCCAGCTCCATTGCAAA-3'
XPO4-F	5'- GGAGGCTGCAGGAAGATAAGG-3'	XPO4-R	5'- CATACACACGTGTTTCAGTTCTTTACC-3'

Primers for qChIP Validation of HDAC1 High-Ranked Peaks (Supplementary Figure 2a)

CCNA2-F	5'-CAGACGGCGCTCCAAGAG-3'	CCNA2-R	5'-GGGCGCTGCCTTTTCC-3'
CREG1-F	5'-CGTTGCAGCGTCCCAGAT-3'	CREG1-R	5'-GGAGGCTTGTGGGAAATTCC-3'
EGR1-F	5'-TCCTCACTCGCCCACCAT-3'	EGR1-R	5'-CCCGTTGCTCAGCAGCAT-3'
GPSM2-F	5'-TCGATGCTCAAGAAACCAGAAA-3'	GPSM2-R	5'-GAAGACCTTCCGACAAAAAAGC-3'
H3F3A-F	5'-CGGGACGCTGGATTCCTAT-3'	H3F3A-R	5'-CGCCCCATTGGAAAACG-3'
HHEX-F	5'-GAGGCCTCCAAATGAAACCA-3'	HHEX-R	5'-TCGGATGGCTGGAGTTCAG-3'
HPS3-F	5'-CGAACGTCTGGGCTGTAGCT-3'	HPS3-R	5'-GCAAATTCCGGGAAGCAA-3'
MLXIP-F	5'-GGTCTGAGAAAAATCCTGTCGGTAT-3'	MLXIP-R	5'-TGTCCTCATTATTCTCATCGTATTGAT-3'
NPM3-F	5'-ACGGAGGTGGACGTGTAAATG-3'	NPM3-R	5'-ACCCGCCGTCACGTGTAA-3'
PHF15-F	5'-GGAGCTATCTGCCCTCCTGTCT-3'	PHF15-R	5'-CGGACGTGCTGCGTATCA-3'
PRTN3-F	5'-GAAGAAAACCCCCAGACAAAACT-3'	PRTN3-R	5'-CACCACGAGTGCCCTCAGT-3'
RASA3-F	5'-TGGGCGCCTGCACTTC-3'	RASA3-R	5'-GCAGCGGAGAAAGGGAGAT-3'
UBE2S-F	5'-CCTTCCTCTCTTCTTCCCCTTT-3'	UBE2S-R	5'-GGGTCGGGAGGGTTCTCA-3'
USP39-F	5'-GGAAGCCAGTGCAGGAACAG-3'	USP39-R	5'-GGACAAATCACGCTGCAGAA-3'

ZNF281-F	5'-GCAGGAGGGCAGAGATGGA-3'	ZNF281-R	5'-GAAGACGGTGGTTGAACATAACC-3'

Primers for qChIP Validation of HDAC1 Low-Ranked Peaks (Supplementary Figure 2b)

AAK1-F	5'-CCAGAACTCTGTCTGCACTGAGA-3'	AAK1-R	5'-AGGAGTCGACCGCGAGAA-3'
CLK2-F	5'-GCCACACGTCTCCATGCA-3'	CLK2-R	5'-CCGCCTCCTGTCCTTTCC-3'
EIF2C2-F	5'-AAGGAAAGTCTGGAAATGCTGAA-3'	EIF2C2-R	5'-CCAGGCGACAGCCCTAACTA-3'
GPSM1-F	5'-GGATCCAGCTGGGTTGGAA-3'	GPSM1-R	5'-CCGGAGAGTAGATGCCTTTTCA-3'
HBZ-F	5'-GAGGGAACGATTAGGAGTTGCA-3'	HBZ-R	5'-TGCCTGCCCAGGTCTCA-3'
KDM4C-F	5'-CAGCGAACAGCTGTCACCTAGT-3'	KDM4C-R	5'-CTCCGGCCCAGGGAGAT-3'
KLHDC7B-F	5'-CCTCTGTGCTCTGCATGCA-3'	KLHDC7B-R	5'-GGACCATCTGGCTCCAAGGT-3'
PATZ1-F	5'-TGCTGCACAACCTGAACCA-3'	PATZ1-R	5'-GCAAGAGCACGTCGCAGAA-3'
RCOR1-F	5'-GAGCTTGGGATGAAAATAGCTTCT-3'	RCOR1-R	5'-GGCCGCTGAAAGATGAATGA-3'
TAF1B-F	5'-CGGGAATCCTTGCCACTGT-3'	TAF1B-R	5'-GGAGTTCCCAAACCCGAATT-3'
TK1-F	5'-GGAGAGTACTCGGGTTCGTGAA-3'	TK1-R	5'-AGCACAGTGGGCAGGTTAATG-3'
TNFRSF25-F	5'-GTGTGACTGTGCCGGTGACT-3'	TNFRSF25-R	5'-GGCAGCCTCTGCAACAAAA-3'
TP53INP2-F	5'-CGCCCGGTAGACCATGAC-3'	TP53INP2-R	5'-GGCCTTGGTGACGTCTCAA-3'
ZNF334-F	5'-ACTTGGACCTGGTGCGAACT-3'	ZNF334-R	5'-GCCAGCCCTTCCTATAGGTTGT-3'

Primers for qChIP Validation of $H3K4Me^3$ High-Ranked Peaks (Supplementary Figure 3a)

ABL1-F	5'-GCGGGTGCTTCCTTACCA-3'	ABL1-R	5'-CCCGGCACGACCTTTTAAAT-3'
CCNA2-F	5'-CAGACGGCGCTCCAAGAG-3'	CCNA2-R	5'-GGGCGCTGCCTTTTCC-3'
CDKN2C-F	5'-GCACGTTCAATTCCGAAAGAC-3'	CDKN2C-R	5'-CCGAGCCCTGCAGTTCAC-3'
DYRK2-F	5'- GAACTTCCTCCTTGCTGCTTTC-3'	DYRK2-R	5'-GAAGGGAACTCTGGGATGCA-3'
PIM3-F	5'- GTGCACCGCGACATTAAGG-3'	PIM3-R	5'-CACCCGAACCGAAGTCGAT-3'
STK24-F	5'-GAGACTTGGTTGCATTTACAGGAA-3'	STK24-R	5'-AGGTGTCACCAAGTACCGAAAGA-3'

Primers for qChIP Validation of $H3K4Me^3$ Low-Ranked Peaks (Supplementary Figure 3b)

IL4R-F	5'- CCCTGGTTCAAAGTGAGAAGTGA-3'	IL4R-R	5'-CGTGTAGTCAGATGATGGTGTCTAGA-3'
IL26-F	5'-CAGGCGAGGGCCACAA-3'	IL26-R	5'-TGCATTGCCCACACAAGCT-3'
ING3-F	5'-GCCTGGCACTTCTTGAAACC-3'	ING3-R	5'-CACCTGTTCTTTGATGTGATTCCA-3'
TAGAP-F	5'- GGATGGGAGAGTGGTGAGTTG-3'	TAGAP-R	5'-GGCTAGTATGGCATCTCTGTTCTG-3'
YY1-F	5'- TGGGCGTGGCCTCACT-3'	YY1-R	5'-GGCTCAATCAATGCTCCTGAA-3'

Primers for qChIP Validation of $H3K27Me^3$ High-Ranked Peaks (Supplementary Figure 4a)

BAI1-F	5'- GGGTGAAGGTGGTGCATCTG-3'	BAI1-R	5'- ATGCCCCTCCAAGTGCAA-3'
FLI1-F	5'-CGTGCGCCTGTTTGCAT-3'	FLI1-R	5'- CGCATACCCGCACACAAC-3'

HDAC4-F	5’-CCAGGAAGCTGGCACAGTTT-3’	HDAC4-R	5’-TGACAAAGTGCTGGCCTTCA-3’
MAPK11-F	5’-GGTCCTGCGCAAGAAAGC-3’	MAPK11-R	5’- CGGCACTGGATAGGAAAATAAGC-3’
PPDPF-F	5’- ACCCCACTTCGCTTCTCTCA-3’	PPDPF-R	5’-AGAAGCTGGCCCACCAATG-3’
STK32C-F	5’- CCTGCACTGGGCATTTTCA-3’	STK32C-R	5’-CAGCAGCCACGCTACACTTG-3’

Primers for qChIP Validation of H3K27Me^3 Low-Ranked Peaks (Supplementary Figure 4b)

CD81-F	5’-CTGTACCTCATCGGCATTGCT-3’	CD81-R	5’-CCGCCCGCTCACCAT-3’
EBF1-F	5’-CCTGGCTAACAGGGTGAAACC-3’	EBF1-R	5’-ACCACGTCCGGCTAATTTTTT-3’
MAPK12-F	5’-CCACCACGCCTGGCTAAT-3’	MAPK12-R	5’-CCAGTCTGCCCAACATAGTGAA-3’
PTPRM-F	5’- GGCATCAGGCTGCAGTTGTA-3’	PTPRM-R	5’- GGGCTCCAGTGTTTGTTTGG-3’
STK39-F	5’- CCCTTGCTTATATCCAAACATTTTC-3’	STK39-R	5’-TTCCAGATAGGGAGAGCAATGG-3’

Primers for qChIP Validation of H3K36me^3 High-Ranked Peaks (Supplementary Figure 5a)

CD79B-F	5’-TGGAAGAGTCCCAGAACGAATC-3’	CD79B-R	5’-CAAACCGGATGCCTTGGAT-3’
H3F3B-F	5’-TGTGTGCCATCCACGCTAA-3’	H3F3B-R	5’-CGAGCCAACTGGATGTCTTTG-3’
HLA-C-F	5’-TCCTTTCCCAGAGCCATCTTC-3’	HLA-C-R	5’-GCCAGGCCAGCAACGAT-3’
MIR142-F	5’-GGGCGCCAGCCTCTTC-3’	MIR142-R	5’-AAGGCTATCCCAACAAAAATGG-3’
MYC-F	5’-CTGGAGAGATTTGGGAGCTCAT-3’’	MYC-R	5’-GGGATGGGAGGAAACGCTAA-3’
PCBP1-F	5’-TGGCGTGCCGCAGTCT-3’	PCBP1-R	5’-CCAGCATGACCAGGCAAAT-3’

Primers for qChIP Validation of H3K36me^3 Low-Ranked Peaks (Supplementary Figure 5b)

BCCIP-F	5’-CATGGTGTGGGTATGTGATCAAT-3’	BCCIP-R	5’-CATTCAAGGCATAGAGCCAAAGT-3’
MIRLET7F1-F	5’-GGGCACTGTGTGAGCCAAA-3’	MIRLET7F1-R	5’-CCCAAAACCCAAACTGGAAA-3’
NLRC5-F	5’-GAGAGTGCGGCGATGCAT-3’	NLRC5-R	5’-CGGCTGCTGGCCATTCTA-3’
ORAI2-F	5’-TTTTCCACCTCCAGCACCTT-3’	ORAI2-R	5’-AGCCCCCCATAATTGAAAATG-3’
PDE4DIP-F	5’-ACAGCTGCCATGGTTCAGAAG-3’	PDE4DIP-R	5’-GGCACAACCACGGCATCT-3’
TCF3-F	5’-TGAGGAGGACTCCCAGGACTT-3’	TCF3-R	5’-GCCCCAGCTCTGCAATGT-3’

Primers for qChIP Validation of H3K9Me^3 High-Ranked Peaks (Supplementary Figure 6a)

AJAP1-F	5’- GAAAGACGATCTCACACACAGCAT-3’	AJAP1-R	5’- GGGAATTATTACGCAGGTTTGC-3’
BCL9L-F	5’-CAAGGTCACGCCACTTCCTT-3’	BCL9L-R	5’-AAGGATCGACCCCATTTCAAC-3’
KIF21B-F	5’-GGCCATTCCCCACCAGAT-3’	KIF21B-R	5’-CCATGACCTCCCCAGAGACA-3’
PVRL1-F	5’-AGCCTCATGGTTACCCAAGCT-3’	PVRL1-R	5’-CCCACCTCCCCCTTGACT-3’
SLC25A22-F	5’- TGGCACCCAGCTAGCTCTTC-3’	SLC25A22-R	5’- CATGCTGGGCCACAAAAAA-3’
ZMIZ1-F	5’- AGTTGCTGTGTCCTGGGAGTCT-3’	ZMIZ1-R	5’- CTGAGGCACAGGAATTGACAAG-3’

Primers for qChIP Validation of H3K9Me^3 Low-Ranked Peaks (Supplementary Figure 6b)

MUC6-F	5'-AACCCCATCACACCCTCAGA-3'	MUC6-R	5'-CACCAAGGAGGTGGAGAAAGG-3'
NBPF1-F	5'-GGGAAACCAGCGATTTGGA-3'	NBPF1-R	5'-CCCTCCGCCTGTCTTTCA-3'
PDE4DIP-F	5'- GATCGCCTCAAGTTCTGCATT-3'	PDE4DIP-R	5'- CGCGCCAGAGTCATCGTT-3'
PIK3AP1-F	5'-GGAGATGAGGCCAGATAATAAACC-3'	PIK3AP1-R	5'-GCCTGTTCCTGACCATGGAA-3'
RELT-F	5'- AGGACACCTTCCGGCAGAT-3'	RELT-R	5'-GACAGTCCTGGAGACCCTTGTCT-3'
TMEM72-F	5'-CAGCGCCCCTGAAGGAA-3'	TMEM72-R	5'- CACACATAGCCCTGGCAGAA-3'

Primers for qChIP Validation of H3K9ac High-Ranked Peaks (Supplementary Figure 7a)

ESRRA-F	5'-AAGCTCTGGAGGTGTTTTCATGA-3'	ESRRA-R	5'-ACTCTCACTAGGTCTCCCCACATC-3'
HNRNPC-F	5'-CCTTTTTTCGCTCTTCCTGTGT-3'	HNRNPC-R	5'-CCTCGCGGTCGTAGAAAATG-3'
NFE2L1-F	5'-TCGCATCAGCTGCGTTAAAA-3'	NFE2L1-R	5'-TGGCGGCCGGATAACC-3'
RAB4A-F	5'-TTCGGTTTCAGCTGCGATATT-3'	RAB4A-R	5'-CAGGCGTCGCCCTAAGC-3'
SH2D4B-F	5'-GATCATAGGGAACACAGCCAAC-3'	SH2D4B-R	5'-GGCACGGCTGCTTTGAAC-3'
TPP1-F	5'-GGGAGGGTGGGAACACAAG-3'	TPP1-R	5'-GGCAGCAGGTGGGTTTCA-3'

Primers for qChIP Validation of H3K9ac Low-Ranked Peaks (Supplementary Figure 7b)

JUNB-F	5'-GGGCTCTGGGTTCCCTCATA-3'	JUNB-R	5'-CCCTCCCCGAGTCAGAGAA-3'
MYST2-F	5'-GGCCCCCAGTGGTCTCA-3'	MYST2-R	5'-GGCGAGTGCCCACTTCCTA-3'
PINX1-F	5'-TGAGGGCAGGTCACGTCTTT-3'	PINX1-R	5'-CTCCATCAGGAAGCCCTAGGT-3'
TAF12-F	5'-CTTAACGCCGGGTTGGAA-3'	TAF12-R	5'-TTGTGGTTGAGTGGCAGCAT-3'
THAP9-F	5'-GGTCCGATTCTGGCTGGAT-3'	THAP9-R	5'-CCACTGTCGAGCCAAATGAA-3'
VCPIP1-F	5'-AAATCAAATGGCGCCTCAGT-3'	VCPIP1-R	5'-TCTCGGAAGCTCTGCCTTCTC-3'

qChIP Analysis of Epigenetic Changes at Ikaros-only and Ikaros-HDAC1 Target Genes
(Figure 2, Supplementary Figures 11-13)

Primers for serial qChIP experiments spanning the transcription start sites (TSS) of Ikaros-only and Ikaros-HDAC1 target genes (5 primer pairs each gene) are shown below.

CDC2-F1	5'-GTAAGAAAGAAAGAGAGAAAGAAGG-3'	CDC2-R1	5'-CATCATATCCTTGAGAGAGATTTCTGCAC-3'
CDC2-F2	5'-GAGGTAGAAACAAAGCACAGCG-3'	CDC2-R2	5'-CAGTGACCTTAAGAAGGAAATG-3'
CDC2-F3	5'- TTCCTCTTTCTTTCGCGCTCTA-3'	CDC2-R3	5'- AGCCAATCAGAGCCCAGCTA-3'
CDC2-F4	5'-GACTGGAGGTCAGGGATCTGCG-3'	CDC2-R4	5'-CACACGGCCAGACCCAGGCGAAGGCCCTG-3'
CDC2-F5	5'-GAACTCGTGGGCCTTCTTGGGCTTG-3'	CDC2-R5	5'-GTGCCTGGGCTGACTGTTCTTAAC-3'
CDC7-F1	5'-CTATTTCCTCTGCTTCCACACTTG-3'	CDC7-R1	5'-CAAGGGAGTAATATGATCTATC-3'
CDC7-F2	5'-CTCAAGTAGTAATCAATGCCGGAAAG-3'	CDC7-R2	5'-CTGTAGGAAGCTAAATTCCAATGAAG-3'

CDC7-F3	5’-CTGGAAATCATGCTGGGCCCTTAG-3'	CDC7-R3	5'-CTAGAAGAGCGGTTACGGGAATGAAG-3'
CDC7-F4	5’-GAACGAATTCTGGATCGTGACTTC-3'	CDC7-R4	5'-CTTTCCCGCCGCAGGTCGGAGGGATC-3'
CDC7-F5	5’-CTCGCCGTTTCCCACTGCGCCTGCTG-3'	CDC7-R5	5'-CTGTCAGAAACACCGGCAGGGGGCTG-3'
ANAPC1-F1	5’- GAACTCGGGAGGCGGAGGTTGCAG-3'	ANAPC1-R1	5’- CTCGCTCTTTCGCCCAGGCTG-3'
ANAPC1-F2	5’-GTGGAGACAGGGTCTCGCT-3'	ANAPC1-R2	5’-GAGGCGAGGGACAGGATTGCTG-3'
ANAPC1-F3	5’- GCACGGAAGGTGGAAATAGC-3'	ANAPC1-R3	5’- TGCGCTGTTGAGGGAAGAC-3'
ANAPC1-F4	5’-CTGACGCGTCCCGAGTTATACAG-3'	ANAPC1-R4	5’-GAGTCACGCGGCGCGCGACAGAC-3'
ANAPC1-F5	5’-CTCCCGTTAGTGTGCATTTGGGTCTC-3'	ANAPC1-R5	5’-CAGTATCAGCAATGATTCCTAC-3'
ANAPC7-F1	5’-GAGCTTCAACTGTGACAAAATG-3'	ANAPC7-R1	5’-ATGCTCTGAAAAAATCACAGTG-3'
ANAPC7-F2	5’-GTCCCAATAACTTGTGTAAAAC-3'	ANAPC7-R2	5’-AGCTGATACCCATATAAACCAG-3'
ANAPC7-F3	5’- AGGAGCAGGGACTCGAGTTTC-3'	ANAPC7-R3	5’- CACCCCCAAGCAAGAGGTAA-3'
ANAPC7-F4	5’-CGTGAGTTGAGGCGGGTAGCTGCCTG-3'	ANAPC7-R4	5’-CACGTGCCCTGAGCCCCCGCTCCCAG-3'
ANAPC7-F5	5’-ATGCGCTAGCCCCCGCATCTGAG-3'	ANAPC7-R5	5’GTTTCCCAAGGCCAGAAAGTGGAC-3'

References

1. Dovat S, Ronni T, Russell D, Ferrini R, Cobb BS, Smale ST. A common mechanism for mitotic inactivation of C2H2 zinc finger DNA-binding domains. *Genes Dev* 2002 Dec 1; **16**(23)**:** 2985-2990.

2. Gurel Z, Ronni T, Ho S, Kuchar J, Payne KJ, Turk CW*, et al.* Recruitment of ikaros to pericentromeric heterochromatin is regulated by phosphorylation. *J Biol Chem* 2008 Mar 28; **283**(13)**:** 8291-8300.

3. Popescu M, Gurel Z, Ronni T, Song C, Hung KY, Payne KJ*, et al.* Ikaros stability and pericentromeric localization are regulated by protein phosphatase 1. *J Biol Chem* 2009 May 15; **284**(20)**:** 13869-13880.

4. Li Z, Song C, Ouyang H, Lai L, Payne KJ, Dovat S. Cell cycle-specific function of Ikaros in human leukemia. *Pediatr Blood Cancer* 2012 Jul 15; **59**(1)**:** 69-76.

5. Hawley RG, Lieu FH, Fong AZ, Hawley TS. Versatile retroviral vectors for potential use in gene therapy. *Gene Therapy* 1994; **1:** 136-138.

6. Wang Z, Zang C, Rosenfeld JA, Schones DE, Barski A, Cuddapah S*, et al.* Combinatorial patterns of histone acetylations and methylations in the human genome. *Nat Genet* 2008 Jul; **40**(7)**:** 897-903.

7. Fujiwara T, O'Geen H, Keles S, Blahnik K, Linnemann AK, Kang YA*, et al.* Discovering hematopoietic mechanisms through genome-wide analysis of GATA factor chromatin occupancy. *Mol Cell* 2009 Nov 25; **36**(4)**:** 667-681.

8. Cox AJ. ELAND: Efficient large-scale alignment of nucleotide data. 2007.

9. Ji H, Jiang H, Ma W, Johnson DS, Myers RM, Wong WH. An integrated software system for analyzing ChIP-chip and ChIP-seq data. *Nat Biotechnol* 2008 Nov; **26**(11)**:** 1293-1300.

10. Jothi R, Cuddapah S, Barski A, Cui K, Zhao K. Genome-wide identification of in vivo protein-DNA binding sites from ChIP-Seq data. *Nucleic Acids Res* 2008 Sep; **36**(16)**:** 5221-5231.

11. Seal RL, Gordon SM, Lush MJ, Wright MW, Bruford EA. genenames.org: the HGNC resources in 2011. *Nucleic Acids Res* 2011 Jan; **39**(Database issue)**:** D514-519.

12. Song C, Gowda C, Pan X, Ding Y, Tong Y, Tan BH*, et al.* Targeting casein kinase II restores Ikaros tumor suppressor activity and demonstrates therapeutic efficacy in high-risk leukemia. *Blood* 2015 Oct; 126(15):1813-22
